# Evaluation of projection‐ and dual‐energy‐based methods for metal artifact reduction in CT using a phantom study

**DOI:** 10.1002/acm2.12347

**Published:** 2018-05-10

**Authors:** Zaiyang Long, Michael R. Bruesewitz, David R. DeLone, Jonathan M. Morris, Kimberly K. Amrami, Mark C. Adkins, Katrina N. Glazebrook, James M. Kofler, Shuai Leng, Cynthia H. McCollough, Joel G. Fletcher, Ahmed F. Halaweish, Lifeng Yu

**Affiliations:** ^1^ Department of Radiology Mayo Clinic Rochester MN USA; ^2^ Siemens Healthcare Malvern PA USA

**Keywords:** CT, dual energy, iMAR, metal artifact, Mono+

## Abstract

**Objectives:**

Both projection and dual‐energy (DE)‐based methods have been used for metal artifact reduction (MAR) in CT. The two methods can also be combined. The purpose of this work was to evaluate these three MAR methods using phantom experiments for five types of metal implants.

**Materials and Methods:**

Five phantoms representing spine, dental, hip, shoulder, and knee were constructed with metal implants. These phantoms were scanned using both single‐energy (SE) and DE protocols with matched radiation output. The SE data were processed using a projection‐based MAR (iMAR, Siemens) algorithm, while the DE data were processed to generate virtual monochromatic images at high keV (Mono+, Siemens). In addition, the DE images after iMAR were used to generate Mono+ images (DE iMAR Mono+). Artifacts were quantitatively evaluated using CT numbers at different regions of interest. Iodine contrast‐to‐noise ratio (CNR) was evaluated in the spine phantom. Three musculoskeletal radiologists and two neuro‐radiologists independently ranked the artifact reduction.

**Results:**

The DE Mono+ at high keV resulted in reduced artifacts but also lower iodine CNR. The iMAR method alone caused missing tissue artifacts in dental phantom. DE iMAR Mono+ caused wrong CT numbers in close proximity to the metal prostheses in knee and hip phantoms. All musculoskeletal radiologists ranked SE iMAR > DE iMAR Mono+ > DE Mono+ for knee and hip, while DE iMAR Mono+ > SE iMAR > DE Mono+ for shoulder. Both neuro‐radiologists ranked DE iMAR Mono+ > DE Mono+ > SE iMAR for spine and DE Mono+ > DE iMAR Mono+ > SE iMAR for dental.

**Conclusions:**

The SE iMAR was the best choice for the hip and knee prostheses, while DE Mono+ at high keV was best for dental implants and DE iMAR Mono+ was best for spine and shoulder prostheses. Artifacts were also introduced by MAR algorithms.

## 1 INTRODUCTION

The prevalence of metal arthroplasty and implants has been increasing in the United States.^1^, ^2^, ^3^ More than 7 million Americans were reported to have total knee arthroplasty or total hip arthroplasty in 2014.^1^ Total shoulder arthroplasty procedures increased by about 5% annually between 1993 and 2007 and were predicted to further increase.^3^ A wide range of spine instrumentation has been used for various clinical indications such as trauma, tumors, and degenerative disk disease.^4^ Dental implants have also become ubiquitous nowadays both for health and cosmetic reasons.^5^ Medical imaging procedures, including computed tomography (CT), are frequently performed in all of these populations for planning, treatment guidance, or diagnosis. For example, the degree of osseous fusion is often evaluated using CT for imaging bony details in the spine.^4^ Unfortunately, metal prostheses and implants are often associated with substantial artifacts in medical imaging. In CT, the metals can cause severe artifacts in a form of streaking or shadowing throughout the images due to a number of issues, including beam hardening, photon starvation, noise, scattering, and nonlinear partial volume effect. The appearance of these artifacts varies significantly, depending on metal composition, size, and orientation, as well as CT acquisition parameters. These artifacts often significantly undermine radiologist diagnostic performance and confidence.

Extensive research efforts have been devoted to metal artifact reduction (MAR) in CT.^6^, ^7^, ^8^, ^9^, ^10^, ^11^, ^12^ One major theme is to identify the metal implant‐corrupted region in the projection data and replace the affected data using different inpainting/interpolation methods. Another is to use statistics‐based and/or model‐based iterative reconstruction by segmenting the metal implant‐corrupted region and utilize prior knowledge of the imaging physics, system geometry, and noise properties to improve reconstruction quality. All major manufacturers have developed their own proprietary metal artifact reduction techniques. For example, one of the major CT manufacturers combines normalized metal artifact reduction (NMAR) and frequency‐split metal artifact reduction (FSMAR) strategies working in projection and image spaces in an iterative fashion (iMAR, Siemens Healthcare, Germany).^7^, ^13^ NMAR involves metal segmentation, computation and forward‐projection of artifact‐free prior images, normalization of the original sinogram by the prior sinogram, and interpolation. Therefore, it avoids direct interpolation which could generate additional artifacts due to unsmooth transition between original and direct‐interpolated projection data. FSMAR makes uses of the high‐frequency information of the original images and low‐frequency information of NMAR‐corrected images. Consequently, a spatially weighted sum is generated in order to maintain edges and fine anatomical structures as well as low level of noise. The scope of this work will focus on the different MAR methods from this specific manufacturer. Some technical details of the MAR methods from other manufacturers can be found in a few previous publications, such as Huang et al. and Andersson et al.^14^, ^15^ Clinical performances using these commercially available techniques vary to some extent, especially for different metal implant types.^14^, ^15^, ^16^, ^17^, ^18^, ^19^, ^20^

In addition to the abovementioned MAR methods, virtual monochromatic images generated from dual‐energy (DE) CT is also being used for metal artifact reduction.^21^, ^22^, ^23^, ^24^ Low and high tube voltage scans from DE CT contain different spectral information and allow the synthesis of virtual monochromatic images through basis material decomposition.^25^ Monochromatic images at low energy provide better iodine contrast‐to‐noise ratio, while high‐energy images can minimize metal artifacts because the appropriately chosen weighting factor could cancel out some of the artifacts between the two basis material images. With virtual monochromatic images, beam‐hardening artifacts can be reduced while other factors such as scatter and photon starvation could still remain to affect the images. This approach was shown to provide promising metal artifact reduction effects for different metal prostheses or implants.^24^, ^26^, ^27^, ^28^, ^29^

Recently, the combination of projection‐based and DE‐based methods becomes available. However, the efficacy of the combination method has not been reported in the literature. Therefore, the aim of this study was to systematically evaluate three MAR methods (iMAR, virtual monochromatic imaging, and the combination of the two methods), in comparison to single energy without iMAR and DE linearly mixed images for the task of metal artifact reduction using a phantom study.

## 2 MATERIALS AND METHODS

### 2.A Phantoms and experimental setup

Five phantoms (spine, dental, hip, shoulder, and knee) were constructed to evaluate five popular types of metal arthroplasty and implants:

1. A three‐dimensional (3D)‐printed spine model with pedicle screws was constructed using a 3D printer (Objet 350 Connex3, Stratasys, Eden Prairie, MN, USA). The spine model was placed in a cylindrical water tank of 35 cm × 25 cm × 50 cm to mimic the attenuation level of an average sized patient. Two syringes containing iodine solutions (Ultravist, Berlex Inc., Montville, NJ, USA) were placed on both sides of the spine model in the phantom to allow evaluation of iodine contrast‐to‐noise ratio (CNR).
2. A dental model with metal fillings was fixed to the bottom of a human skull without the mandible and placed in a 25 × 19 × 19 cm cuboidal water tank to mimic the patient attenuation of head.
3. A set of hip metal prostheses (Zimmer Inc., Warsaw, IN, USA) were placed adjacent to a human pelvic bone and one tibia in z 35 cm × 25 cm × 50 cm cylindrical water tank. A tibia was used instead of femur due to the limited length of the water tank.
4. A knee metal prosthesis (Zimmer Inc., Warsaw, IN, USA) was placed adjacent to a human tibia in a 17 × 27.5 × 15 cm cuboidal water tank.
5. A shoulder metal prosthesis (3M Neer‐II, Maplewood, MI, USA) was placed in the glenoid cavity of a custom anthropomorphic phantom.

### 2.B CT scans

Phantoms were scanned on a dual‐source CT scanner (SOMATOM Definition Flash, Siemens Healthcare, Erlangen, Germany) using both single‐energy (SE) and DE protocols with the same volume CT dose index (CTDIvol) for each phantom type. Scan parameters were kept as close as possible to our routine clinical exam protocol with reconstruction kernels matched between SE and DE scans. The detailed scan and reconstruction parameters can be found in Table 1. For CNR comparison in the spine phantom, additional SE images at tube potentials of 80, 100, and 140 kV were also acquired with the same CTDIvol. All reconstructions were performed using an iterative reconstruction (SAFIRE, Sinogram‐Affirmed Iterative Reconstruction, Siemens Healthcare, Erlangen, Germany) at a strength level of 3.

**Table 1:** Scan and reconstruction parameters for the five phantoms with metal implants or prostheses

Phantoms	DE			SE			CTDIvol (mGy)	Slice thickness/gap (mm)
	Tube voltage (kV)	Collimation (mm)	Recon kernel	Tube voltage (kV)	Collimation (mm)	Recon kernel		
Spine	100/Sn140	32 × 0.6	Q30	120	128 × 0.6	Q30	11.5	0.75/0.7
Dental	100/Sn140	32 × 0.6	Q30	120	128 × 0.6	Q30	11.3	1/0.1
Shoulder	80/Sn140	40 × 0.6	Q30	140	128 × 0.6	Qr40	11.2	1/0.6
Hip	100/Sn140	32 × 0.6	Q30	140	128 × 0.6	I40	15.8	1/0.8
Knee	80/Sn140	40 × 0.6	Q30	120	128 × 0.6	Qr40	15.8	0.6/0.3

### 2.C Data processing

**Figure 1:**
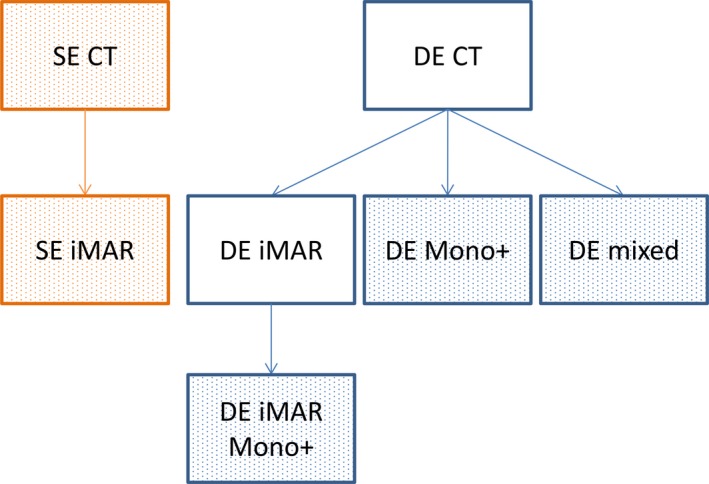
Different types of images can be generated from either single‐energy or dual‐energy scans. Five main methods, three with metal artifact reduction including SE iMAR, DE Mono+, and DE iMAR Mono+, as well as two without including SE and DE mixed, were evaluated and compared (filled boxes).

All SE data were processed using iMAR with respective implant setting, that is, spine, dental, shoulder, hip, and knee settings. Monochromatic images at 130 keV were synthesized from DE CT using commercially available software (Mono+, Syngo.Via, Siemens Healthcare, Erlangen, Germany).^30^ In addition to iMAR and Mono+, a combined method was also used to reduce metal artifacts. In this combined method, the DE raw data were processed first using iMAR for both the low‐ and high‐kV scans, and then the reconstructed low‐ and high‐kV images were loaded to the Mono+ software to generate the Mono+ images at 130 keV. This combined method is referred to as DE iMAR Mono+. Therefore, each phantom generated a total of five sets of images, including three sets with artifact reduction (SE iMAR, DE Mono+, DE iMAR Mono+), DE images mixed with a ratio of 0.5 between the low‐ and high‐kV images (DE mixed) and SE images (Fig. 1).

### 2.D Data analysis

**Figure 2:**
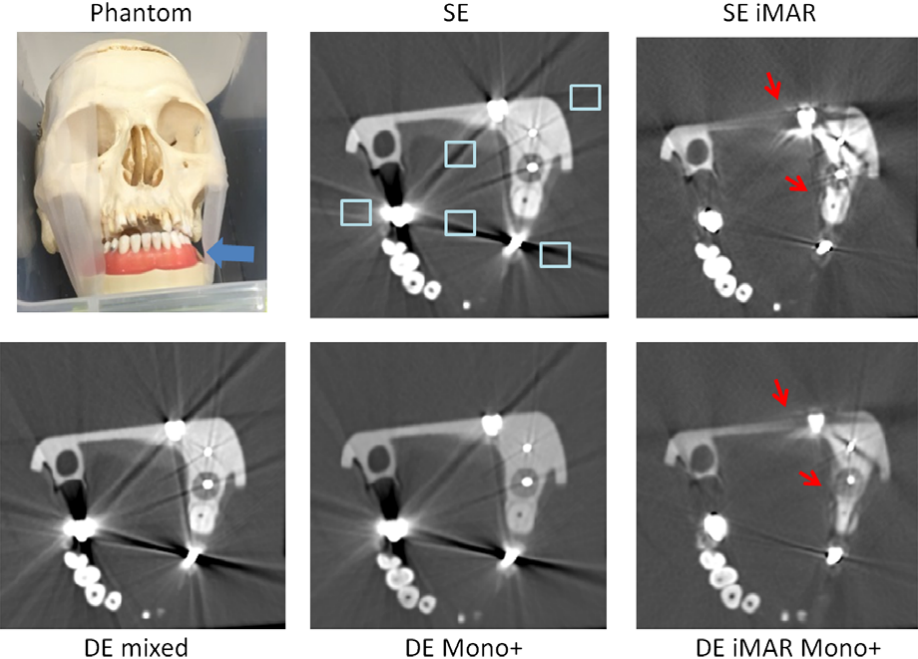
Phantom setup and representative SE, SE iMAR, DE mixed, DE iMAR, and DE iMAR Mono+ images for the dental phantom study. Blue arrow points to the dental model with the implants. Red arrows point to missing tissue artifacts that were observed. Positions of the artifact regions of interest (light blue box) are shown in the SE images.

**Figure 3:**
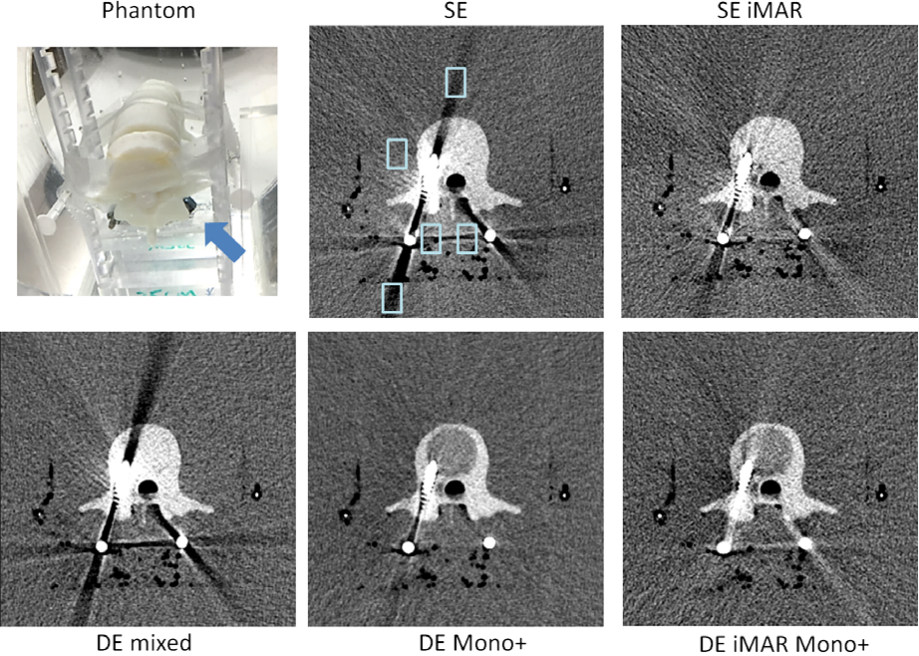
Phantom setup and representative SE, SE iMAR, DE mixed, DE iMAR, and DE iMAR Mono+ images for the spine phantom study. Blue arrow points to the 3D‐prined spine model with pedicle screws. Positions of the artifact regions of interest (light blue box) are shown in the SE images.

**Figure 4:**
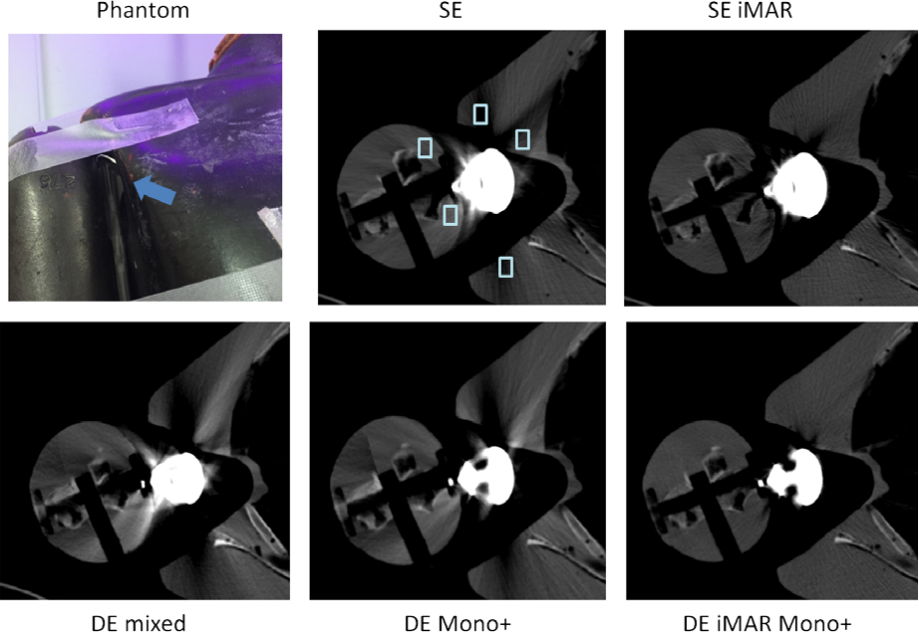
Phantom setup and representative SE, SE iMAR, DE mixed, DE iMAR, and DE iMAR Mono+ images for the shoulder phantom study. Blue arrow points to the shoulder prosthesis placement. Positions of the artifact regions of interest (light blue box) are shown in the SE images.

**Figure 5:**
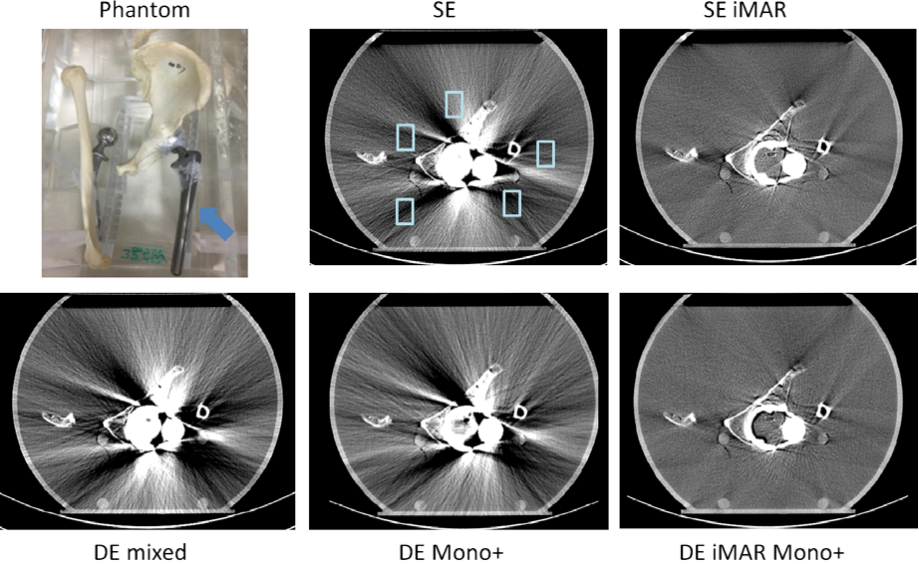
Phantom setup and representative SE, SE iMAR, DE mixed, DE iMAR, and DE iMAR Mono+ images for the hip phantom study. Blue arrow points to the hip prostheses placement. Positions of the artifact regions of interest (light blue box) are shown in the SE images.

**Figure 6:**
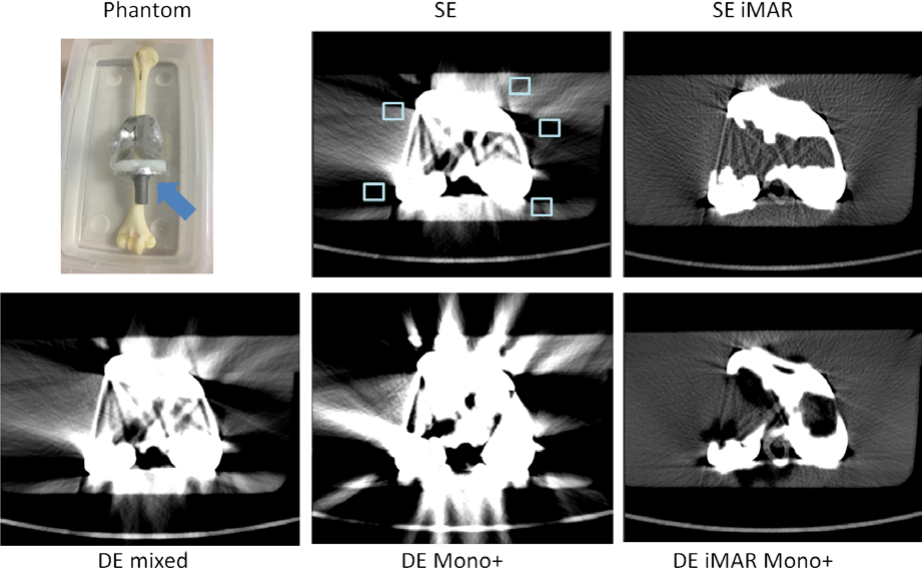
Phantom setup and representative SE, SE iMAR, DE mixed, DE iMAR, and DE iMAR Mono+ images for the knee phantom study. Blue arrow points to the knee prostheses placement. Positions of the artifact regions of interest (light blue box) are shown in the SE images.

For each image series obtained using the five methods, three axial slices containing metal artifacts were identified. Five regions of interest (ROI) in the water or tissue‐mimicking background near the metal (artifact ROIs) and two ROIs of the same size in background regions without metal contamination (reference ROIs) were placed and populated through all three slices for each phantom. The size and placement of the artifact ROI can be seen in Figs. 2, 3, 4, 5, 6. The CT number and standard deviation in each ROI were measured. The mean absolute differences between the artifact and reference ROIs were depicted using box‐and‐whisker plots and used as a quantitative measure of artifact severity.

Furthermore, the spine and dental images with metal artifact reduction (SE iMAR, DE iMAR, DE iMAR Mono+) were independently and blindly reviewed and ranked by two experienced neuro‐radiologists (Experience years: 10 and 20) for the overall metal artifact reduction and image quality. Similarly, shoulder, hip, and knee images were independently and blindly reviewed and ranked by three experienced musculoskeletal (MSK) radiologists (experience years: 20, 22, and 24) for the overall metal artifact reduction and image quality. Consensus was reached through discussion if the initial ranking was not exactly the same. Attention was paid to whether there was new artifact introduced by the metal artifact reduction methods, because it is known that certain MAR methods can introduce new artifacts.^14^, ^31^

In addition, we also evaluated the impact of metal artifact reduction on the CNR for the spine phantom. The mean CT numbers of the iodine solution and background noises were measured, and iodine CNR was calculated across ten slices of all the SE iMAR (80, 100, 120, and 140 kV) and DE Mono+ images (50, 70, 130, and 190 keV) of the spine phantom.

## 3 RESULTS

**Figure 7:**
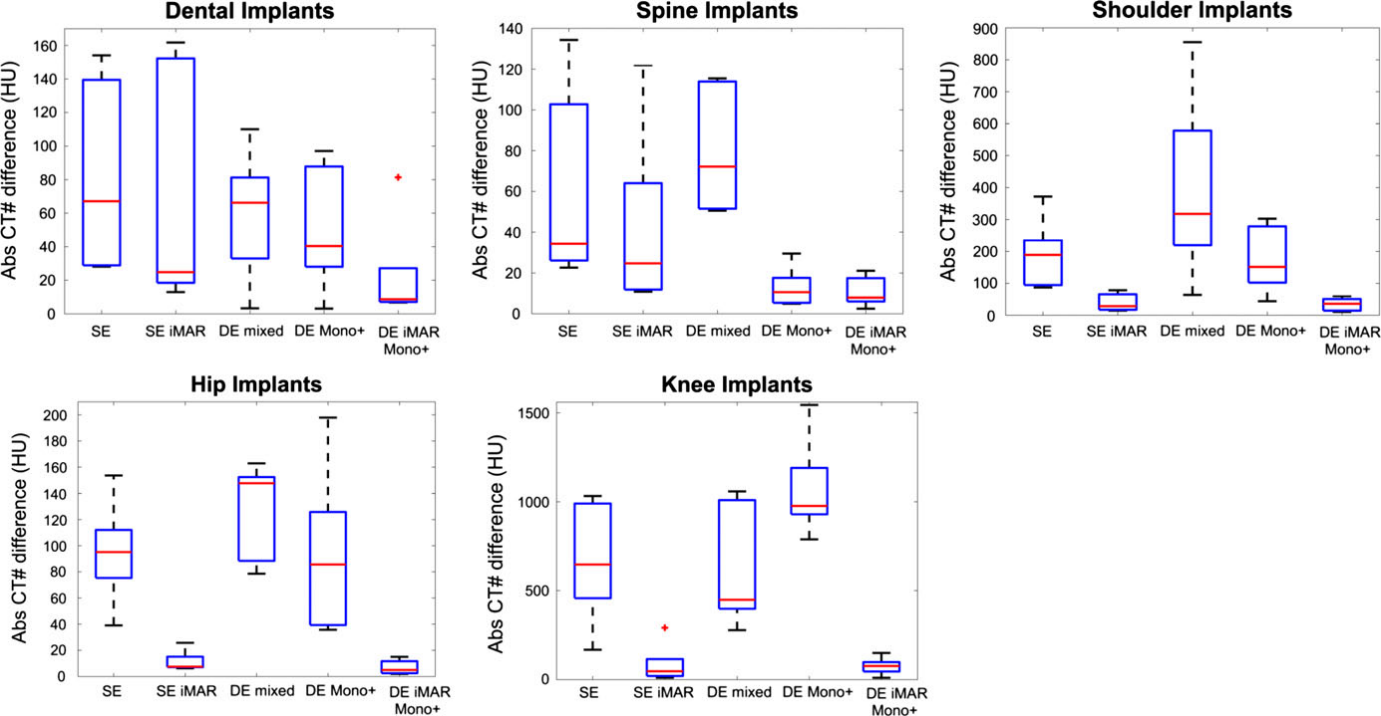
Boxplots for background artifact analysis for SE, SE iMAR, DE mixed, DE iMAR, and DE iMAR Mono+ images of all five phantoms.

Phantom setup and representative image comparisons for all methods are shown for each phantom in Figs. 2, 3, 4, 5, 6. Figure 7 demonstrated the quantitative background artifact reduction performance of all methods for all phantom settings. For dental metal implant, the original SE, SE iMAR, DE mixed, DE Mono+, and DE iMAR Mono+ showed median CT number differences between the artifact and artifact‐free ROIs of 67.1, 24.8, 66.2, 40.0, and 8.7, respectively. For the spine phantom, the corresponding CT number differences were 34.3, 24.7, 72.2, 10.6 and 7.9, respectively.

For shoulder, the original SE, SE iMAR, DE mixed, DE Mono+, and DE iMAR Mono+ showed median CT number differences of 189.0, 28.4, 317.2, 151.3, and 35, respectively. For the hip prostheses, the corresponding values were 95.1, 7.5, 147.7, 85.6, and 4.9, respectively. For the knee, the corresponding values were 647.0, 46.9, 448.7, 976.2, and 76.5, respectively.

For the blind image reviewing and ranking, the two experienced neuro‐radiologists both ranked DE Mono+ > DE iMAR Mono+ > SE iMAR for dental implants and DE iMAR Mono+ > DE Mono+ > SE iMAR for spine implants (Table 2). Missing tissue was observed in images reconstructed with iMAR for dental implants (Fig. 2). All three experienced MSK radiologists ranked DE iMAR Mono+ > SE iMAR > DE Mono+ for the shoulder prosthesis and SE iMAR > DE iMAR Mono+ > DE Mono+ for the knee and hip prostheses.

**Table 2:** Blinded image review and ranking results from two experienced neuro‐radiologists and three musculoskeletal radiologists based on overall metal artifact reduction and image quality

	1st	2nd	3rd
Dental	DE Mono+	DE iMAR Mono+	SE iMAR
Spine	DE iMAR Mono+	DE Mono+	SE iMAR
Shoulder	DE iMAR Mono+	SE iMAR	DE Mono+
Hip and knee	SE iMAR	DE iMAR Mono+	DE Mono+

**Figure 8:**
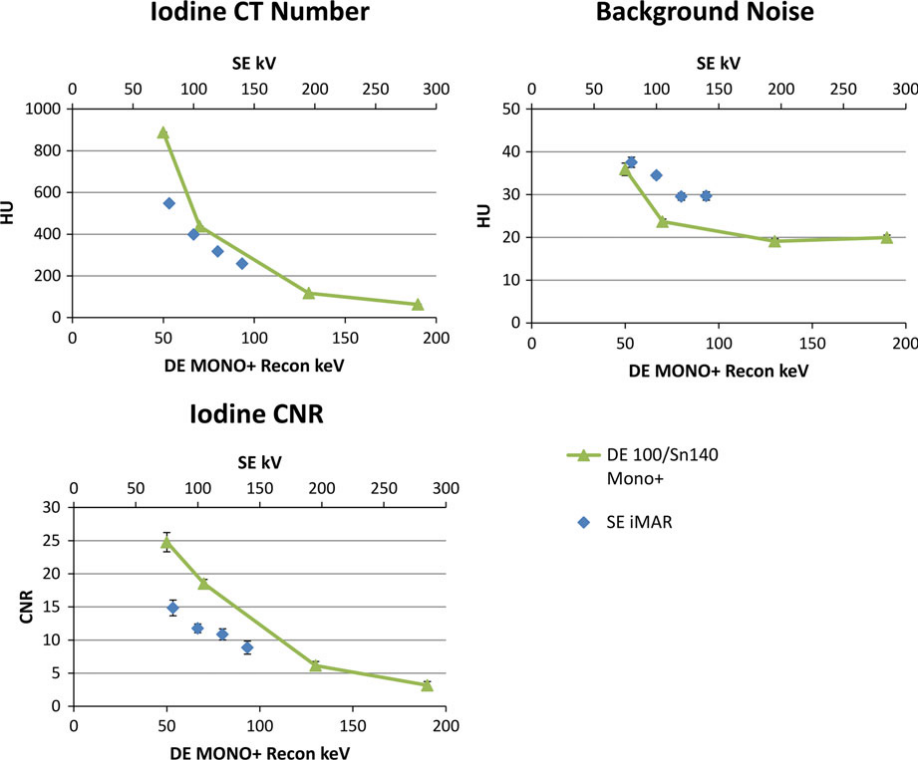
Plots for Iodine contrast, background noise, and iodine CNR between SE iMAR at different tube potentials and DE Mono+ at different keV for the spine phantom.

In addition, iodine CNR values from DE Mono+ at high keV, for example, above 130 keV, were lower compared to those from SE iMAR at 80–140 kV. The iodine CNR results from DE Mono+ at various virtual monochromatic energies (50–190 keV) and SE iMAR at different tube potentials (80–140 kV) in the spine phantom were plotted in Fig. 8.

## 4 DISCUSSION

With the availability of both projection‐based (e.g., iMAR) and dual‐energy‐based (e.g., Mono+) MAR methods, as well as the ability to combine the two methods (DE iMAR Mono+), radiologists or technologists will need to decide which technique to use for a patient with metal implants. If one scans with a SE technique, then the only option available for metal artifact reduction is iMAR. When a DE scan technique is used, one would have to decide whether to use iMAR, Mono+, or both. This phantom study evaluated and compared three MAR methods (Siemens iMAR, Mono+, and DE iMAR Mono+) together with the original SE and DE mixed images for five types of metal implants and prostheses.^28^ The results from this study can provide guidance for radiologists and technologists with regard to the most appropriate technique on this manufacturer's platforms.

As mentioned above, other CT manufacturers have their own proprietary algorithms, that is, virtual monochromatic imaging from fast kV‐switching dual‐energy data with or without MAR from GE (GE Healthcare, Milwaukee, WI, USA), MAR algorithm for orthopedic devices (O‐MAR) from Philips (Philips Medical Systems, Best, The Netherlands), and single‐energy metal artifact reduction (SEMAR) from Toshiba (Toshiba Medical Systems, Otawara, Japan). Streakings and noise levels were demonstrated to reduce to different extents using these algorithms, while sometimes new artifacts were introduced.^14^, ^15^, ^22^, ^32^ Huang et al. and Andersson et al. studied the combination of virtual monochromatic images with MAR algorithms on the Discovery CT750 HD (GE Healthcare, Milwaukee, WI, USA) scanners, with one investigating three types of metal implants and the other focusing on one type of implant.^14^, ^15^ Similar to our findings, Huang et al. also demonstrated that different strategies showed different extent of metal artifact reduction for dental, hip, and spine implants.^14^ No universal method worked the best for all types of metal implants or prosthesis. This is not unexpected because of the different size, geometry, and material composition of the different metal implants.

Causes of metal artifacts are complex, including beam hardening, nonlinear partial volume effects, increased scatter and noise. To reduce these artifacts, the optimal acquisition and reconstruction method needs to be prescribed for patients with specific types of metal prostheses and implants. DE iMAR Mono+, combining iterative metal artifact reduction in projection and image spaces with virtual monochromatic imaging, showed the best artifact reduction for the spine prosthesis. The quantitative artifact analysis of DE iMAR Mono+ images demonstrated the median CT number difference closest to 0. The neuro‐radiologists also ranked it the highest in terms of overall image quality. On the other hand, DE Mono+ was a better choice for dental implants, because iMAR (SE iMAR and DE iMAR Mono+) caused missing tissues artifacts (Fig. 2). As described above, iMAR involves metal segmentation using a simple threshold for each implant setting.^20^ For a dental implant, it might be difficult to obtain a correct segmentation due to the dental anatomy. For the shoulder prosthesis, quantitative artifact analysis revealed similar performance between SE iMAR and DE iMAR Mono+. The MSK radiologists ranked DE iMAR Mono+ the highest because it better delineated the implant shape. Therefore, DE iMAR Mono+ was chosen to be the best choice for shoulder prosthesis. Finally, for hip and knee prostheses, quantitative artifact analysis showed that SE iMAR had median CT number differences closest to 0; while DE iMAR Mono+ also performed well, it caused wrong CT numbers in proximity to the metal, especially around the knee prosthesis and along inner edge of the hip prosthesis (Figs. 5 and 6). This artifact could be due to the nonideal weighting factor used to cancel metal artifact from the DE iMAR images. Therefore, SE iMAR worked the best for hip and knee prostheses. Overall, metal artifact in the knee phantom was the hardest to correct because of the relatively large size of the prosthesis compared to the surrounding tissue.

As demonstrated in the spine phantom, iodine CNR is reduced with increased virtual monochromatic energy for DE images. High monochromatic energies, for example, 130 keV, which demonstrate the best metal artifact reduction, lead to reduced iodine CNR compared to SE images acquired with 80–140 kV. Low monochromatic energies result in better iodine CNR values but have no metal artifact reduction effect. The energy levels were sampled from 50 to 190 keV in this study. Further studies should be performed to explore the optimal energies to achieve sufficient iodine CNR and metal artifact reduction results for specific clinical tasks.

The current study has several limitations. First, the MAR methods evaluated were from one CT vendor. Comparison of MAR methods with other vendors would provide interesting information in the future. Second, there were some differences between the SE and DE scans in terms of acquisition techniques, such as detector configuration. The SE scans use 128 × 0.6 mm (physical collimation 64 × 0.6 mm with a z‐flying focal spot), while the DE scans use either 32 × 0.6 mm or 40 × 0.6 mm, which could lead to slight differences in scattering and noise. Third, although we tried our best to obtain a variety of metal implants, only limited numbers were available to us for our study. In addition, the exact composition of these implants was not available. Literature provides some insights^33^, ^34^, ^35^; however, there are still a lot of relevant materials including metal alloys, ceramics, and polymers, with a wide range of densities. Finally, because of certain limitations of the phantoms, that is, pedicle screws cannot be removed from the spine phantom and metal fillings cannot be removed from the dental phantom, we could not acquire metal artifact‐absent reference images for artifact quantification. Evaluations of patient data with metal implants or prostheses is needed to confirm these phantom study results.

## 5 CONCLUSION

This phantom study investigated metal artifact reduction effects of SE iMAR, DE Mono+, and DE iMAR Mono+, in comparison to SE and DE mixed images, on five types of metal prostheses and implants. SE iMAR was demonstrated to be the best choice for the hip and knee prostheses, DE Mono+ at high keV for dental implants and DE iMAR Mono+ for spine and shoulder prosthesis in this study.

## CONFLICT OF INTEREST

Cynthia McCollough receives industry funding from Siemens Healthcare. Ahmed Halaweish is an employee of Siemens Healthcare. The remaining authors have no conflict of interest.
